# Contrasting surface warming of a marginal basin due to large-scale climatic patterns and local forcing

**DOI:** 10.1038/s41598-020-74758-7

**Published:** 2020-10-19

**Authors:** Naomi Krauzig, Pierpaolo Falco, Enrico Zambianchi

**Affiliations:** 1 Department of Science and Technology, Parthenope University, Centro Direzionale, Isola C4, 80143 Naples, Italy; 2 CoNISMa, Piazzale Flaminio 9, 00196 Rome, Italy; 3 ISMAR-CNR, Via Fosso Del Cavaliere 100, 00133 Rome, Italy

**Keywords:** Physical oceanography, Climate-change impacts

## Abstract

The Mediterranean Sea is one of the first regions where sea surface temperature (SST) increase was linked to greenhouse effects and global warming. Due to its sensitivity to climate variability and its high impact on local and remote climate conditions, much effort has been made to assess the SST variability in the Mediterranean as a whole. However, the Mediterranean is composed of several basins, each of which plays a different role in its conveyor belt’s function. This study focuses on the basin of the Tyrrhenian Sea which represents one of the crucial areas for deep mixing of the Mediterranean main water masses. Thirty-seven years (1982–2018) of satellite-derived data were used to investigate the SST variability in relation to large-scale and local forcing mechanisms. A significant warming trend of 0.034 ± 0.004 °C/year was found, which led to an average warming of 1.288 ± 0.129 °C over the considered period. The observed warming presents time-dependent spatial patterns as well as changes in the seasonal cycle. Our results highlight that the Tyrrhenian’s individual long-term surface variability has different characteristics than the Mediterranean as a whole and provide insight into the relative influence of large-scale teleconnection patterns and local air-sea interaction on this variability.

## Introduction

SST is considered a major climatic status indicator, quantifying climatic variability and warming in the oceans^1^. The Fifth Assessment Report of the IPCC^2^ highlighted a long-term intensifying trend in global SST due to climatic change and contemporary atmospheric warming.

Sea surface warming has been suggested to be directly driven by atmospheric processes^3^ through increases in downward longwave radiation due to greenhouse gases, which are further amplified by the water vapor feedback and atmospheric adjustment. On the other hand, SST variability has also been linked to variations in the heat transport such as wave-induced thermocline changes, or a decrease in upwelling which is related to a slowdown of wind-driven Ekman pumping^4,5^.

A different study^6^ indicates that SST variability on longer timescales results from a combination of both atmospheric and oceanic processes. They indicated that SST is driven by intrinsic modes of atmospheric circulation variability that imprint themselves upon the SST field mainly via surface energy fluxes and also by oceanic processes such as upwelling, entrainment, and lateral advection. Due to the thermal inertia of the upper-ocean mixed layer, low-frequency atmospheric variability influences SST anomalies more efficiently than synoptic variability^7^. This low-frequency circulation variability tends to be organized into recurring large-scale patterns in particular geographical regions (i.e. teleconnections).

The Mediterranean Sea has proven to be particularly sensitive to global warming^8–10^. Different SST warming trends (ranging from ~ 0.15 to ~ 0.06 °C/year)^8–16^ have been found in this basin depending on the time period considered, specific geographical area and methodology.

In a warming climate, rising SSTs appear to influence the weather and to amplify the magnitude and frequency of extreme events^15–19^ which in turn impact communities and ecosystems^20,21^. Therefore, it is significant to start focussing our attention on individual sub-basins, especially those with very high population density along their surrounding coasts. Among the Mediterranean’s basins, the Tyrrhenian is the most populated semi-enclosed one^22^, thus one of the most potentially vulnerable^23^. Furthermore, the response of the individual sub-basins to climate forcing can contribute to a much better understanding of the general response of the whole Mediterranean. The fact that the Tyrrhenian Sea represents the main area of deep mixing for waters from the Eastern and Western Mediterranean^24^ adds further significance to the investigation of this particular basin.

In addition, even though the variability of the SST has been studied extensively in the Mediterranean Sea, a specific evaluation for the Tyrrhenian Sea has not been carried out. To the best of our knowledge, this basin has only been marginally included in a clusterized SST investigation concerning the Mediterranean Sea^15^. Therefore, we analyzed the SST variations within the Tyrrhenian Sea over the last 37 years by taking advantage of the longest available high resolution satellite-derived dataset from the Copernicus Marine Environment Monitoring Service (CMEMS).

Among the variety of available SST data, this dataset^25^ provides the longest record of foundation temperature with high spatial and temporal resolution^16^ (further details in “Methods”) which has been shown to be suitable for studying SST trends and anomalies at regional scales^11^ with a bias less than 0.1 K^2﻿6﻿^.

## Results

### Observed surface warming in the Tyrrhenian Sea

The temporal and spatial SST variability in the Tyrrhenian Sea was investigated based on the satellite-derived SST dataset from the Copernicus Marine Environment Monitoring Service (see Methods). The overall spatially-averaged warming trend from 1982 up to the beginning of 2019 was 0.034 ± 0.004 °C per year (at least *p* < 0.05 for all presented trends) with a total warming of 1.288 ± 0.129 °C for the 37-year period. This trend ranges spatially from a minimum value of 0.026 °C/year to a maximum of 0.044 °C/year (details in Supplementary Information (SI), Fig. S1(a)), which account for total SST increases between 0.962 and 1.628 °C across the basin.

**Figure 1:**
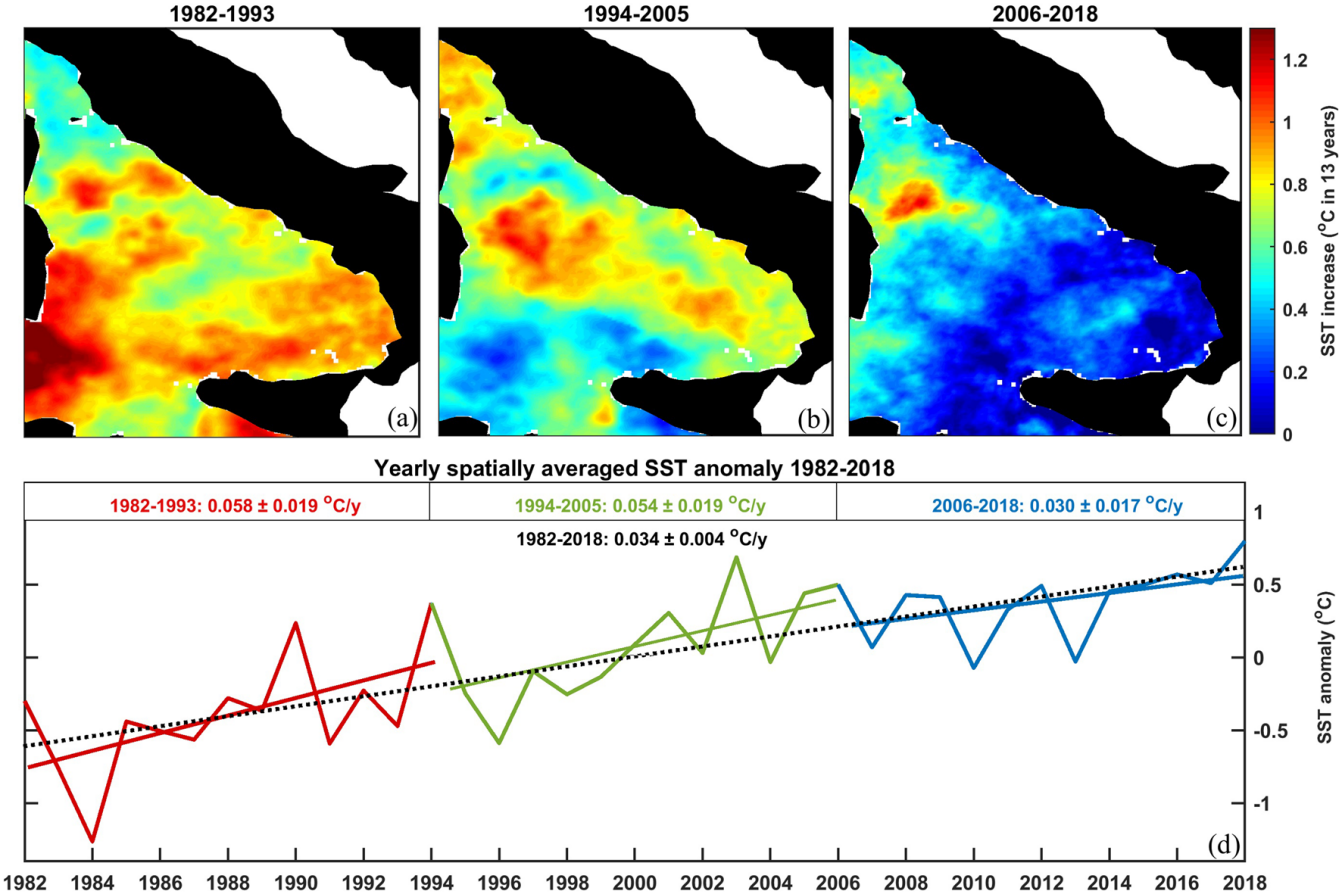
Spatial distribution of the warming trend during (**a**) 1982–1994, (**b**) 1994–2006 and (**c**) 2006–2018; (**d**) yearly values of the spatially-averaged SST increase with trends per individual periods (color solid lines) and trend for the whole time span (black dotted line). The graphical representations were conducted using MATLAB 2019a (https://it.mathworks.com/downloads/web_downloads/download_release?release=R2019a).

Warming trends were not uniform throughout the different time periods (Fig. 1). To be more precise, very low increase rates were observed in more recent years, which differ significantly from those regarding the Mediterranean Sea (Table S1). Based on the observed changes of the spatially-averaged trends, we divided the 37-year time series into three periods of approximately 12 years, showing that the warming trends were also spatially inconstant throughout the basin. For the first period (1982–1993) a warming rate of 0.058 ± 0.019 °C/year accounted for a strong SST increase across almost the entire basin, with a horizontal westward increasing gradient. This gradient changed during the second period (1994–2005) where a slightly lower warming trend led to higher SST in the central and the northern part of the basin and to a significant lower increase rate in the southern sector.

The last period (2006–2018) was characterized by a much lower trend of 0.030 ± 0.017 °C/year with almost constant SST values in the southeastern basin of the Tyrrhenian Sea (Fig. 1c). The average trend remained positive, but SST increases over 1 °C were restricted east of the Bonifacio Strait, indicating that during this period the (cold-core) wind-induced circulation structure known as North Tyrrhenian Cyclone^27^ experienced more significant warming than the rest of the basin.

Moreover, seasonal and monthly analyses were performed to investigate the intra-annual warming consistency. The trend was clearly stronger during spring (0.042 ± 0.003 °C/year) and summer (0.052 ± 0.004 °C/year) than autumn (0.026 ± 0.004 °C/year) and winter (0.021 ± 0.002 °C/year). The spring/summer warming trends were not only higher but also had different spatial patterns (see Fig. S1), than autumn/winter, indicating that the Tyrrhenian Sea presents important time-dependent spatial variability. The overall SST trend was dominated by significant increases during the warm seasons, which lead to phenology changes in the seasonal cycle (Fig. 3, Fig. S2). More specifically, summer transition and duration metrics showed significant trends towards earlier summer onsets (− 0.658 ± 0.153 days/year) and later summer ends (+ 0.222 ± 0.146 days/year), resulting in an extended duration of 34.079 ± 8.301 warm summer days over the entire 37-year period (+ 0.921 ± 0.224 days /year).

### Local air–sea interaction and surface dynamics

Complex patterns of SST variability in the Tyrrhenian Sea show significant local dependence and temporal fluctuations, indicating that governing processes act across a wide range of temporal and spatial scales. This investigation was based on ECMWF ERA5 atmospheric data (see “Methods”).

The best fitting multiple linear regression demonstrated that a significant part (60.8%, *p* < 0.001) of the monthly de-seasoned SST variability can be associated with variations (Table 1) of air temperature (T_air_), net shortwave heat flux (SWHF), total cloud coverage (TCC), mean sea level pressure (MSLP), wind speed (U), zonal wind component (u_10_), zonal wind stress (τ_x_) and the associated Ekman transport (M_Ex,_ M_Ey_) and pumping (M_Ez_).

**Table 1:** Analyzed parameters with their annual trends and their maximum correlation coefficient with SST at the corresponding time lag in months (*p* < 0.05 in bold).

Parameter	Unit	Annual trend	SST correlation	Lag
T_air_	°C	**0.027**	**0.690**	**0**
NHF	W m^−2^	− *0.126*	− **0.254**	**1**
SWHF	W m^−2^	**0.138**	**0.344**	**0**
LWHF*	W m^−2^	**0.029**	− *0.126*	*–*
SHF*	W m^−2^	− **0.027**	− *0.137*	*–*
LHF*	W m^−2^	− **0.268**	− **0.356**	**1**
TCC	%	− **0.012**	− **0.154**	**0**
MSLP	N m^−2^	− **2.700**	**0.196**	**0**
MF*	kg m^−2^ s^−1^	− **4.890 × 10**^**–8**^	− **0.326**	**1**
E*	m of water	− **9.241 × 10**^**–6**^	− **0.357**	**1**
E-P-R	m of water	− **1.674 × 10**^**–5**^	− **0.251**	**1**
U	m s^−1^	**0.002**	− **0.419**	**0**
u_10_	m s^−1^	**0.005**	− **0.229**	**0**
v_10_	m s^−1^	− *0.001*	*0.091*	*–*
τ	N m^−2^	**2.345 × 10**^**–5**^	− **0.196**	**0**
τ_x_	N m^−2^	**2.590 × 10**^**–5**^	− **0.173**	**0**
τ_y_	N m^−2^	− *4.459* × *10*^*–6*^	− *0.118*	*–*
M_Ez_	m s^−1^	− **6.685 × 10**^**–10**^	**− 0.183**	**0**
M_Ex_	m^2^ s^−1^	*− 1.399* × *10*^*−4*^	**0.132**	**0**
M_Ey_	m^2^ s^−1^	**− 3.639 × 10**^**−4**^	**0.186**	**0**

The positive lag refers to a lagging response (of 1 month) to SST forcing. Note that parameters with * are negative values based on the heat and freshwater budget definition.

On the other hand, the lead-lag cross-correlation with net heat flux (NHF), latent heat flux (LHF), evaporation (E), freshwater budget (E-P-R) and moisture flux (MF) indicated that the coupled variability is characterized by a lagging response (of 1 month) to SST forcing.

The estimated relation of the mentioned atmospheric and oceanic parameters with the SST variability was investigated further through the relative magnitude and shapes of monthly and seasonal correlations, whereas spatial trend distributions gave further necessary indications about the potential physical mechanisms. Further details are available in the Supplementary Information (Fig. S3–S7, Table pointed out significant correlations).

### Large-scale atmospheric and oceanic teleconnection patterns

Regional climate trends cannot be understood without considering the impact of variations in large-scale atmospheric circulation^28^. Large-scale atmospheric circulation variability can be characterized by teleconnection patterns, which feature circulation anomalies related to each other at large distances. In order to examine the impact of different teleconnection patterns (data from the NOAA Climate Prediction Centre and from the National Center for Atmospheric Research, see “Methods”) on the Tyrrhenian SST, the normalized time series of the East Atlantic pattern (EA), the North Atlantic Oscillation (NAO) and the Scandinavia pattern (SCAND) were used for correlational analyses with the spatially-averaged SST time series.

Furthermore, several authors^14,16,26,29,30^ pointed out significant correlations between the SST in the Mediterranean Sea and the Atlantic Multidecadal Oscillation (AMO). Therefore, the SST variability in the Tyrrhenian Sea has also been correlated with the AMO index in order to investigate the relationship with the natural oscillation of the SST in the North Atlantic.

Among the selected large-scale atmospheric variability patterns only the EA pattern is significantly correlated with the SST variations (r = 0.654, *p* < 0.001; see Table S3). Even though the NAO index has been proven to have significant impacts on the Mediterranean climate^31–35^, surprisingly no statistically significant correlation with the spatially-averaged SST in the Tyrrhenian basin was found. In particular, the NAO index exhibited very low (negative) and no statistical significant Pearson correlation coefficients (r = − 0.023, *p* = 0.891). Similarly, for the SCAND pattern, despite the impacts on precipitation and temperature fields of the Mediterranean Region^36^, on annual timescales no significant correlations were found, neither with the SST time series nor with any other atmospheric variability pattern.

In order to capture dominant spatio-temporal features of the SST variability, EOF analysis was also applied to the high-resolution satellite-derived dataset. The first three EOF modes accounted for ~ 84% of the total non-seasonal variance of the SST. The spatial amplitude of the dominant (~ 74% of the variance) first mode showed positive values throughout the whole area, indicating an in-phase warming in the entire Tyrrhenian basin (Fig. S8). Details on the results of the EOF analysis can be found in the Supplementary Information (Fig S8–S10).

**Figure 2:**
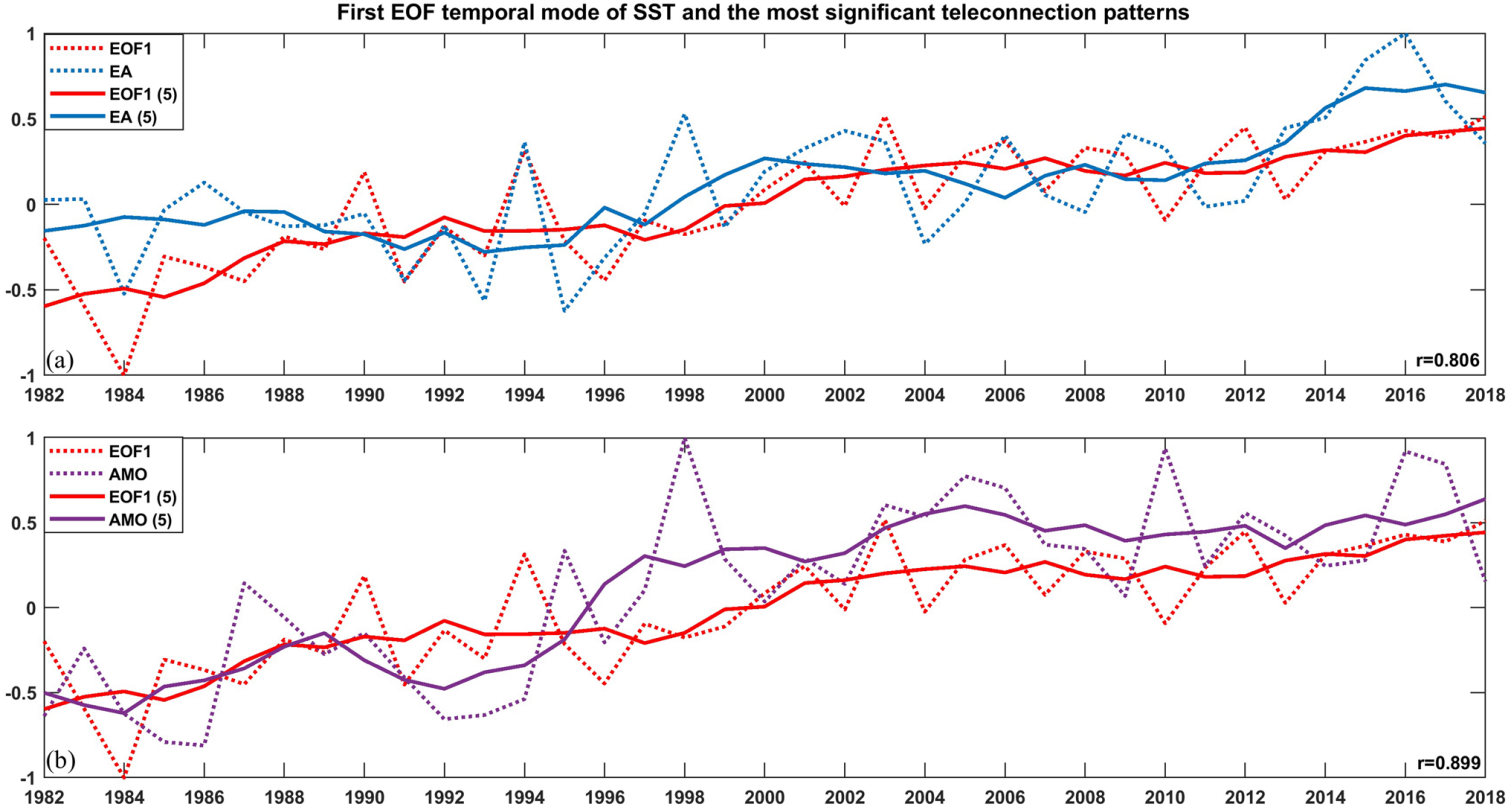
Normalized annual time series of the first EOF mode of SST anomalies (red, dotted line) together with the (**a**) EA pattern (blue, dotted line) and with (**b**) the AMO index (purple, dotted line) during 1982–2018, along with their corresponding 5-year running averages (solid lines).

The link between the leading EOF modes of SST was investigated through correlational analysis with the atmospheric variability patterns (Fig. 2a). The first EOF mode displayed significant correlations with the EA pattern, whereas the second and third temporal modes of SST were not significantly correlated with any of the large-scale variability patterns on annual timescales (Table S3). The analysis also indicated a close relationship between decadal-scale variations of the first EOF temporal mode and the EA pattern (r = 0.806, *p* < 0.001), whereas the other two atmospheric teleconnection patterns are not significantly correlated. The second EOF mode of SST had a slightly significant (negative) correlation exclusively with the EA pattern, whilst the third EOF mode showed significant correlations with the SCAND pattern on those timescales (Table S3).

Furthermore, the correlation between the AMO index and the SST pointed out the link between the sea surface temperature variability in the Tyrrhenian Sea and the oscillation of the SST in the North Atlantic. In particular on decadal timescales, the AMO index displayed remarkably high correlation with the first EOF mode of the SST (r = 0.899, *p* < 0.001, see Fig. 2b). The spatial distribution of the overall warming trend and the correlation coefficient between the SST variability and the AMO also exhibited similar patterns, especially in the southeastern sector and the east coast (SI, Fig. S11).

## Discussion and conclusions

This study investigated the sea surface warming in the Tyrrhenian Sea and its relation to local air-sea interaction as well as large-scale atmospheric and oceanic teleconnection patterns over a period of nearly four decades (1982–2018).

A significant warming trend of 0.034 ± 0.004 °C/year was found, ranging spatially from a minimum value of 0.026 °C/year to a maximum of 0.044 °C/year (Fig. S1(a)) across the basin. The results are in accordance with similar studies regarding the entire Mediterranean basin (see Table S1, in SI).

**Figure 3:**
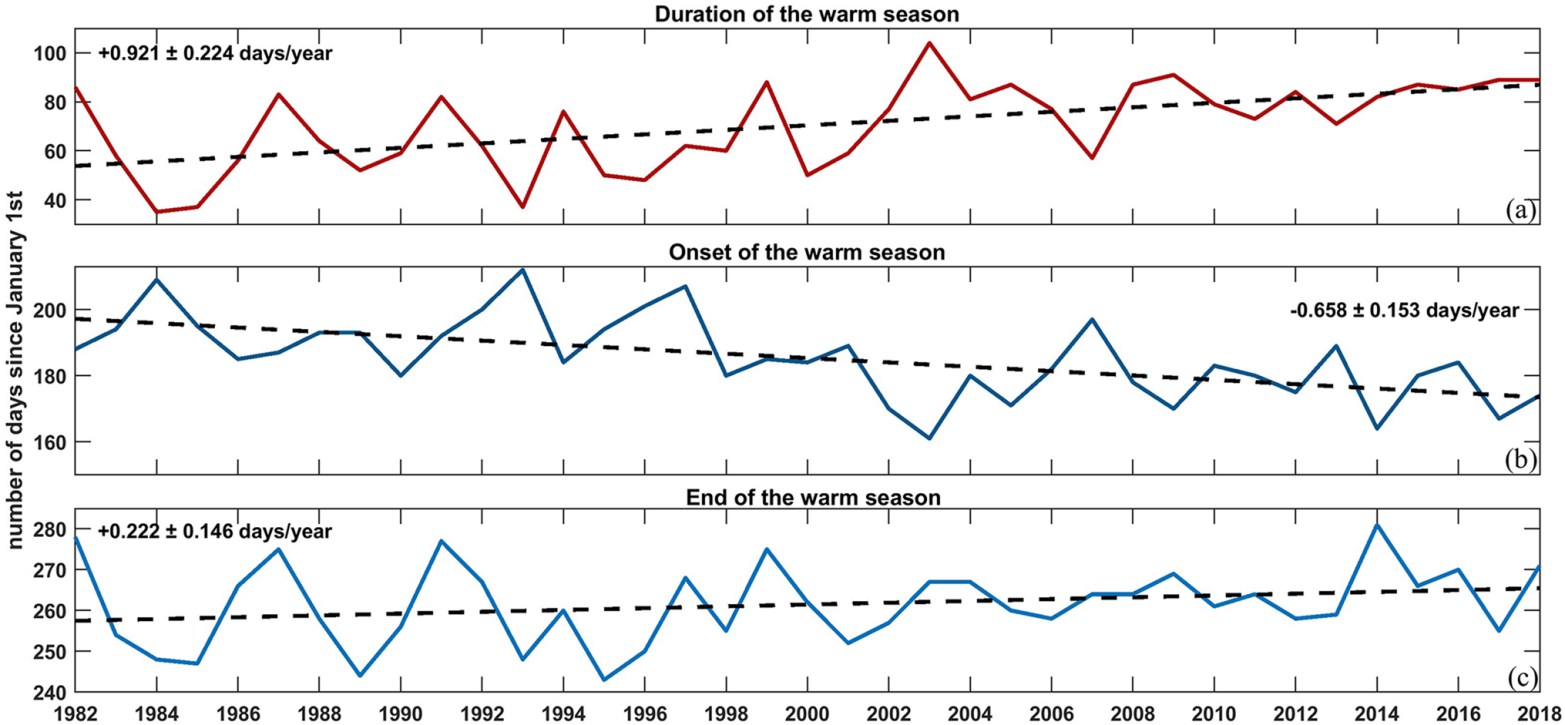
SST phenology changes over the study area for the 37-year study period, showing the trend of (**a**) the number of summer days, (**b**) the summer start date and (**c**) the summer end date.

Separation on monthly and seasonal basis showed that the observed warming was dominated by high increases of SST during the warm seasons. Higher SST trends during spring and summer have also been mentioned recently for the entire Mediterranean Sea^16^. The seasonally differing trends led to SST phenology shifts with a significant tendency towards earlier and longer summer periods (Fig. 3) that are expected to have important ecological implications.

Noteworthy events are the extreme summer SSTs during 2003, which were on average more than 2 °C warmer than the long-term seasonal mean, as well as the ones during July 2006, which led to the highest monthly SST (27.61 °C) ever measured. Indeed, these anomalous episodes have been classified^37^ as strong marine heatwaves with widespread impacts on marine ecosystems and expected subsequent socio-economic consequences^38^.

After 2006 however, the average warming rate of SST in the Tyrrhenian Sea has been found to be slowing down significantly with respect to the past (Fig. 1) and to the rest of the Mediterranean Sea^14,16^.

The physical processes responsible for the SST variability are of both local and hemispheric nature. These processes modulate the long-term variability via large-scale atmospheric and oceanic teleconnections and drive local variations through changes in air-sea interaction. Analysis of the basin surface dynamics and the air-sea energy exchanges indicated that a significant part of the SST variability can be influenced by direct variations of air temperature, shortwave heat flux, total cloud coverage, mean sea level pressure, wind intensity, zonal wind component, zonal wind stress and the associated Ekman transport and pumping. In contrast, the analysis of net heat flux, latent heat flux, evaporation, freshwater budget and moisture flux indicated that the SST potentially drives the air-sea heat flux variability in the Tyrrhenian Sea over the considered period through latent heat loss variations (see Table S2 and Fig. S6–S7).

Summarizing, the SST and T_air_ showed simultaneous significant variations (up to ~ 2012, Fig. S4) which were most likely influenced by the increasing downward solar radiation. Further, in the wind-driven Ekman pumping may affect the spatial distribution of the warming trend. To be more specific, changes in the anticyclonic wind vorticity led to intensified convergence and the spatial distribution of the warming trend is influenced by the resulting Ekman downwelling variability (see Fig. S5). Lastly, latent and sensible heat loss displayed increasing trends, whilst the longwave and the net heat flux were decreasing during 1982–2018. The comparison with the coherent moisture flux and evaporation trend (freshwater budget decreasing trend) supports the finding that the air-sea heat flux variability in the Tyrrhenian Sea is SST-driven.

As for the hemispheric influences, among the selected teleconnection patterns only the EA pattern and the AMO are highly correlated with the long-term warming trend in the Tyrrhenian basin. In general, atmospheric circulation patterns have been proven to impact large-scale SST variations through attendant changes in the turbulent and radiative energy fluxes at the air-sea interface and wind-driven currents^6,39^. In reference to the Mediterranean, the EA pattern has been associated with effects on the net heat budget, the air temperature and the precipitation^40,41^. The combination of these findings suggests that the positive mode of the EA pattern influenced the SST variations through advection of warm air over the basin due to intensification of the low-pressure center and resulted south-westerlies over the Atlantic Ocean.

The suggested linkage between the Mediterranean SST and the AMO agrees with findings from several previous studies^14,16,26,29,30^. However, the absence of lag between the AMO and the Mediterranean SST^23^ differs from the 2-year lag for the Tyrrhenian basin. Besides the remarkably high correlation of the spatially-averaged time series, a strong association also exists between the spatial distributions of the overall warming trend (Fig. S1 (a)) and the correlation coefficient between the Tyrrhenian SST variability and the AMO (Fig. S11). It is worth noticing that the slower warming of the Tyrrhenian Sea after 2006 coincides with the pausing phase of the AMO that has been pointed out recently^16^. Based on these findings, the significant influence of the AMO on the SST tendency should be considered for SST projections, especially since the AMO is expected to shift towards a cold phase in the coming years^42^.

In the Mediterranean, the various and complex interactions between sub-basins form the basis of its conveyor belt mechanism^43^. In particular, the Tyrrhenian Sea is not isolated, as it interacts with the Central Mediterranean in the South^44,45^ and with the Ligurian Sea in the North^46–49^. And yet, as was shown, the response of its individual sub-basins to global warming can be quite different from that of the Mediterranean Sea as a whole. In the case of the Tyrrhenian this response is induced by the varied balance between local and remote forcings, which may yield very contrasting effects. We expect this to be also true for other marginal basins of the world ocean and this study indicates that they need to be investigated separately in order to assess their behavior and their possible vulnerability.

## Methods

### Datasets and processing

The spatio-temporal SST variability during 1982–2018 was investigated utilizing L4 satellite-derived data from the Copernicus Marine Environment Monitoring Service (https://marine.copernicus.eu, accessed 04/2019). The very high spatial (~ 4 × 4 km) and daily temporal resolution data set^25^ was obtained by the Satellite Oceanography Group of the Italian National Research Council (CNR-GOS) through the reanalysis of the Advanced Very High Resolution Radiometer (AVHRR) Pathfinder Version 5.3 (PFV53) data set; effective for studying the Mediterranean SST with a steady bias of less than 0.1 K^23^. This data set covered the period 1981–2014 and has since then been extended and reprocessed by CMEMS, which distribute it freely under the name SST_MED_SST_L4_REP_OBSERVATIONS_010_021 in the CMEMS catalogue. A detailed description of the data processing can be found in the Product User Manual and Quality Information Document that are available in the CMEMS online catalogue^16^.

The relation of the obtained SST variability with the local air-sea interaction was based on monthly time series of atmospheric parameters from the ERA5 dataset, the fifth generation of ECMWF atmospheric reanalysis which is provided by the Copernicus Climate Change Service Climate Data Store (https://cds.climate.copernicus.eu, accessed 05/2019).

The net air-sea exchanges of heat and freshwater were estimated by summing the four heat flux components (net shortwave, net longwave, sensible, latent) and the three freshwater components (evaporation, precipitation, runoff), respectively. The budgets were defined in such a way that positive values indicate heat and freshwater gain for the sea.

Moreover, the wind stress (τ) and the Ekman transport (*M*_*E*_) along with the vertical velocities, which are associated with Ekman pumping, were calculated based on the wind speed (*U*) according to the equations:

1 $$\tau = \rho_{air} C_{d} U^{2}$$

where $\rho_{air}$ = 1.225 kg/m^3^ is the air density, *C*_*d*_ = 1.25 × 10^−3^ is the drag coefficient^50^

2 $$M_{E} = \frac{\tau }{{\rho_{sea} f}}$$

where $\rho_{sea}$ = 1025 kg/m^3^ is the water density and $f = 2\Omega \sin (\theta )$ is the Coriolis parameter with θ = latitude and $\Omega$ = 7.27 × 10^−5^ s^−1^ is the earth’s angular velocity.

The spatio-temporal variability of the mentioned SST and atmospheric data was studied by analyzing the intra-annual and inter-annual geographical and climatological distributions of averages, anomalies and trends within the geographical boundaries of the Tyrrhenian Sea. If not indicated otherwise, all mentioned time series (daily, monthly, seasonal, yearly) were based on the spatial average of this area over the entire 37-year study period.

The intra-annual warming was further investigated analyzing the SST data over the winter (DJF), spring (MAM), summer (JJA) and autumn (SON) months, whereas metrics of SST phenology were used to assess changes in the seasonal cycle. More specifically, summer transition and duration metrics for each year were derived from daily SST data based on the first day and the number of days that exceeded the climatological summer mean, respectively. Hereinafter the duration of the warm summer refers to the length of the within-year period with SST higher than the threshold of 23.688 °C.

In order to obtain the long-term variability the strong seasonal signal was removed from the datasets to form de-seasoned maps and time series. This is achieved by subtracting the climatological monthly mean from each of the specific monthly fields.

Trend estimation and detrending were based on ordinary least squares linear regression. The corresponding uncertainties were defined by standard errors, whereas the statistically significance of the trends was examined through the Mann–Kendall test^51,52^. In this work, the level of significance has been set to α = 0.05 (*p* ≤ 0.05), so that all presented trends are statistically significant at least at the 95% level.

The dominant spatio-temporal features of the SST variability were examined using Empirical Orthogonal Function (EOF) analysis^53^, which was based on the monthly detrended and de-seasonalized SST dataset in order to focus on the non-seasonal modes of variability.

The approximate relation of the mentioned atmospheric and oceanic parameters with the SST was investigated initially by multiple linear regression and lag-lead cross-correlation, whereas spatial trend distributions gave further necessary insight into potential physical mechanisms. Cross-correlation analysis considering the time lag has been shown to indicate the importance of atmospheric forcing versus SST forcing^7,54,55^. Since air-sea interactions are expected to be spatially and seasonally dependent, the lag-lead correlations were analyzed for monthly and seasonally anomalies, taking into account lags from − 6 to + 6 months.

Moreover, the potential impact of low-frequency large-scale variability patterns on the SST was based on normalized time series of the East Atlantic pattern (EA), the North Atlantic Oscillation (NAO), the Scandinavia pattern (SCAND) and the Atlantic Multidecadal Oscillation (AMO). These time series are available from the NOAA Climate Prediction Centre (https://www.cpc.ncep.noaa.gov, accessed 11/2019) and the National Center for Atmospheric Research (https://climatedataguide.ucar.edu, accessed 12/2019), respectively. Details on the teleconnection pattern calculation procedures are freely available^56^ and further information can be found at the NOAA CPC and NCAR websites.

Additional, topographic data covering the area of the Tyrrhenian Sea has been used to represent the surrounding mainland. This dataset is a smoothed one-sixth degree global topography distributed with the jLab^57^ MATLAB package under the name jtopo (https://www.jmlilly.net/doc/about_jtopo.html).

## Supplementary information

Supplementary Information

## Data Availability

The analyzed SST, atmospheric and teleconnection data are freely available through the Copernicus Marine Environment Monitoring Service (https://marine.copernicus.eu), the Copernicus Climate Change Service Climate Data Store (https://cds.climate.copernicus.eu) and the NOAA Climate Prediction Centre (https://www.cpc.ncep.noaa.gov), respectively. Whereas, the topographic dataset is provided freely by Jonathan Lilly (https://www.jmlilly.net/doc/about_jtopo.html).

## Code availability

All data analyses and representations were conducted using MATLAB 2019a (https://it.mathworks.com/downloads). The generated scripts are available upon request from the corresponding author.
