# Supplementary material for: Contrasting surface warming of a marginal basin due to large-scale climatic patterns and local forcing
Source: Sci Rep. 2020 Oct 19;10:17648. doi: 10.1038/s41598-020-74758-7 (PMC7572400; doi:10.1038/s41598-020-74758-7)
Supplement: Supplementary file 1 — Supplementary Information [file 41598_2020_74758_MOESM1_ESM.pdf]

Supplementary Information for

Contrasting surface warming of a marginal basin due to large-scale climatic patterns and local forcing

Naomi Krauzig[1*], Pierpaolo Falco[1,2] & Enrico Zambianchi[1,2,3]

[1] Department of Science and Technology, Parthenope University, Centro Direzionale, Isola C4, 80143 Naples, Italy.
[2] CoNISMa, Piazzale Flaminio 9, 00196 Rome, Italy.
[3] ISMAR-CNR, Via Fosso Del Cavaliere 100, 00133 Rome, Italy.
*email: naomi.krauzig@uniparthenope.it

Contents of this file

Figures S1 to S11

Tables S1 to S3

Introduction

This supplementary file contains further results which support the main text and give a more detailed insight into the surface warming of the Tyrrhenian Sea and its relation to local air-sea interaction as well as large-scale atmospheric and oceanic teleconnection patterns.

Data availability

The analyzed SST, atmospheric and teleconnection data are freely available through the Copernicus Marine Environment Monitoring Service (http://marine.copernicus.eu), the Copernicus Climate Change Service Climate Data Store (https://cds.climate.copernicus.eu) and the NOAA Climate Prediction Centre (https://www.cpc.ncep.noaa.gov), respectively. Whereas, the topographic dataset is provided freely by Jonathan Lilly (https://www.jmlilly.net/doc/about_jtopo.html).

Code availability

All data analyses and representations were conducted using MATLAB 2019a (https://it.mathworks.com/downloads/web_downloads/download_release?release=R2019a). The generated scripts are available upon request from the corresponding author.

Further insight into the observed SST variability

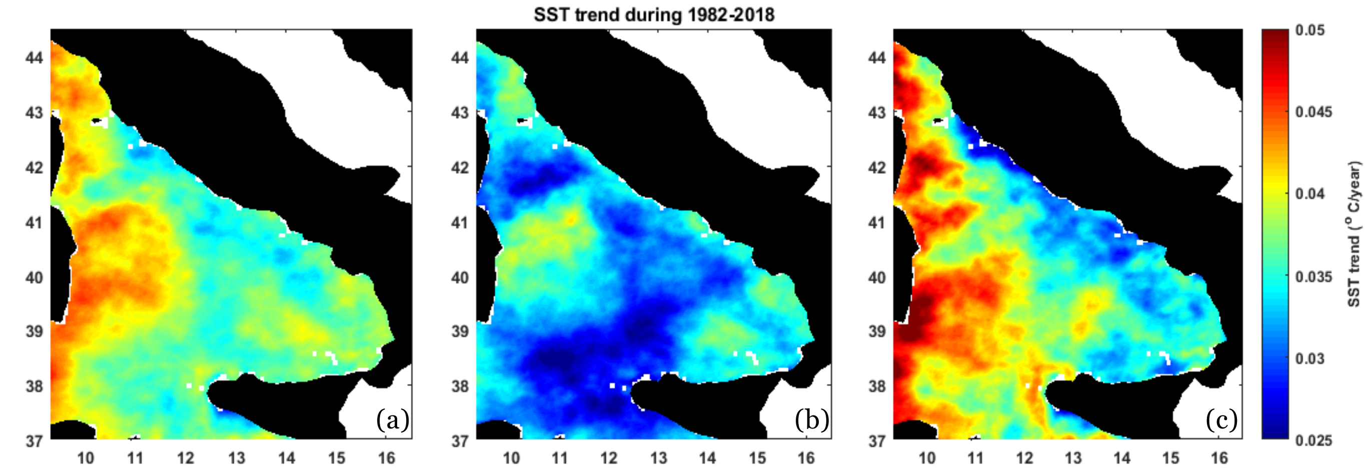

Fig. S1) Spatial distribution of the averaged de-seasoned (a) overall, (b) autumn/winter and (c) spring/summer warming trends during 1982-2018.

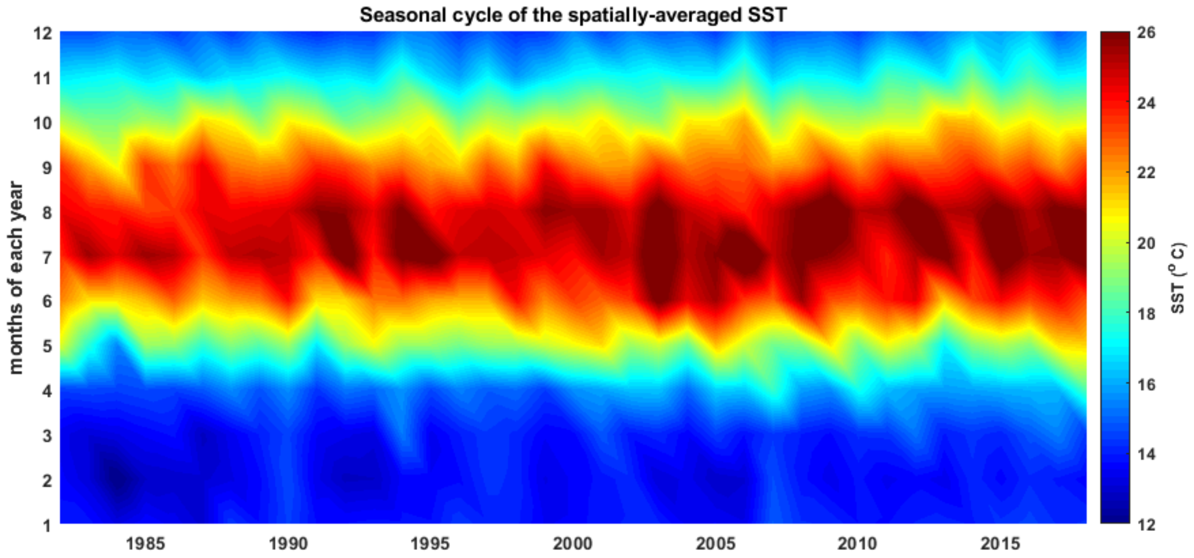

Fig. S2) Hovmöller diagram of SST indicating changes in the seasonal cycle during 1982-2018.

Table S1) Comparison of estimated SST trends in the Mediterranean Sea[1-6] with the ones in this study. All trends for the Tyrrhenian Sea expect the last one (2005-2016) showed consistency through the Mann-Kendall test with 95% confidence intervals. *No standard error estimates are available for this reference.

Period	Mediterranean Sea (°C/y)	Tyrrhenian Sea (°C/y)
1982-1992	0.061 ± 0.019[1]	0.054 ± 0.020
1982-2012	0.035 ± 0.007[2]	0.036 ± 0.005
1982-2016	0.036 ± 0.003[1]	0.033 ± 0.004
1982-2018	0.041 ± 0.006[3]	0.034 ± 0.004
1985-2006	0.040 ± 0.009[4]	0.041 ± 0.008
1985-2008	0.037*[5]	0.038 ± 0.007
1992-2005	0.055*[5]	0.051 ± 0.015
1992-2005	0.060 ± 0.020[6]	0.051 ± 0.015
2005-2016	0.112 ± 0.017[1]	*0.009 ± 0.020*

Local air-sea interaction and surface dynamics

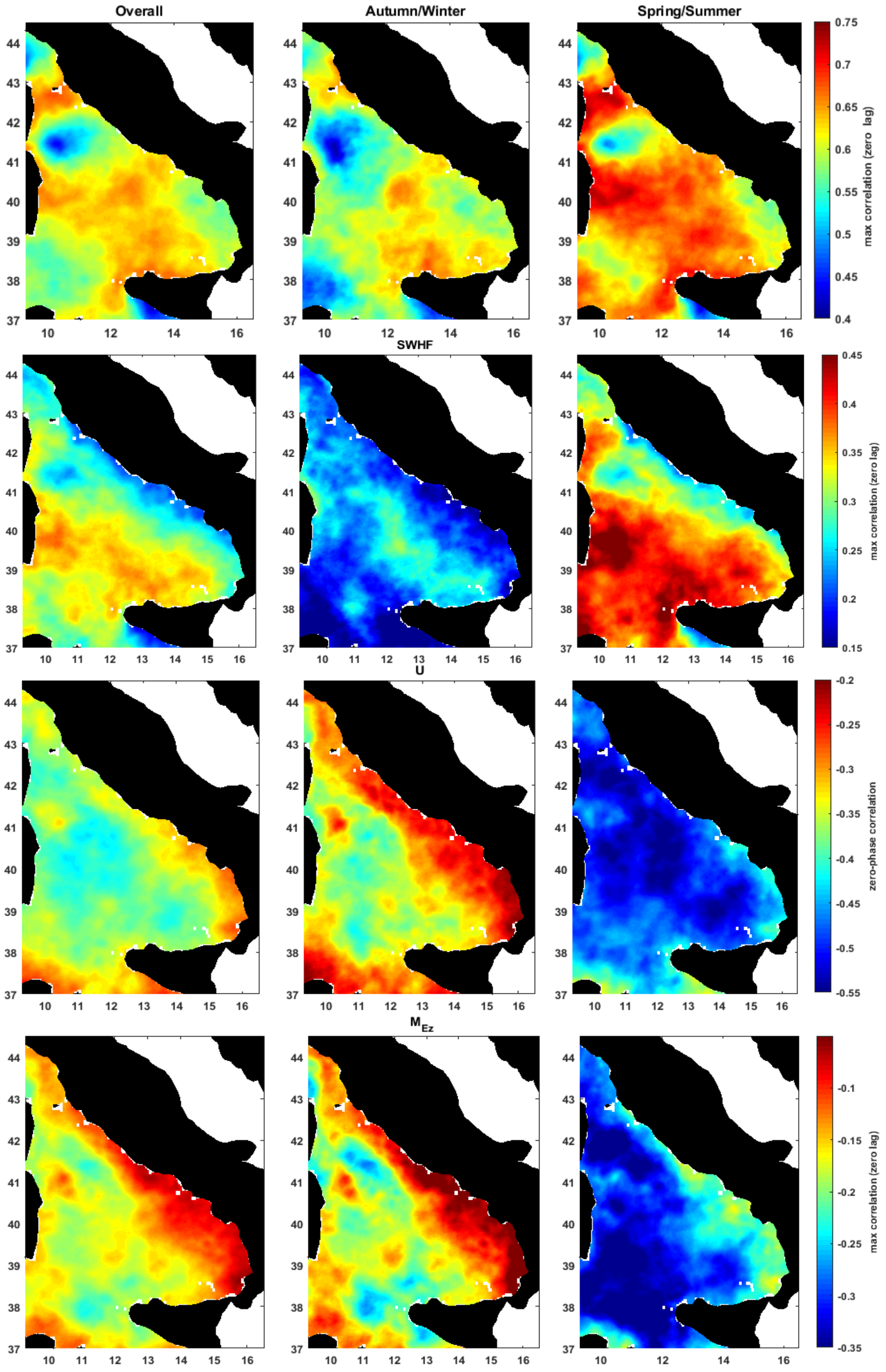

Fig. S3) Distributions of cross-correlation between SST and air temperature (T_{air}), shortwave heat flux (SWHF), wind speed (U) and Ekman pumping (M_{Ez}). Every line consists of three maps; the left is based on the overall time span (1982-2018), the middle one based on autumn/winter periods and the right one is based on spring/summer periods. These maps give insight into the relation of the mentioned atmospheric and oceanic parameters with the SST variability which is discussed in the results and conclusions of the main text.

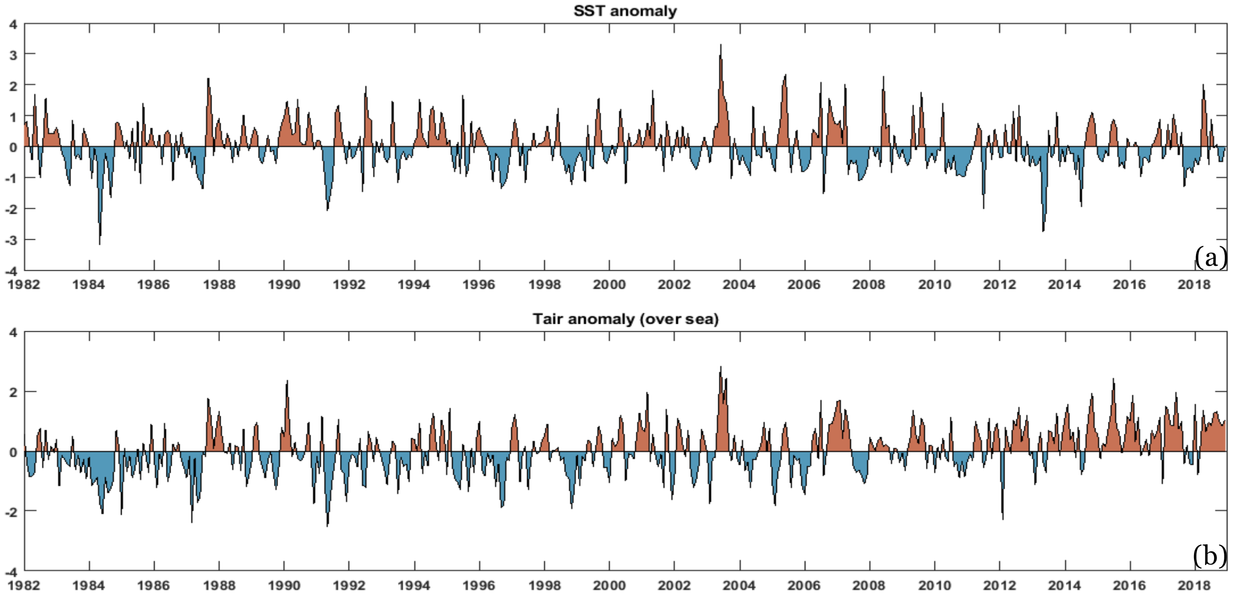

Fig. S4) Simultaneous (a) SST and (b) T_{air} anomalies (in °C) during 1982-2018. The spatially-averaged time series showed significantly higher air temperature anomalies than the SST during the last ~decade, indicating the potential significance of changes in governing atmospheric and oceanic processes.

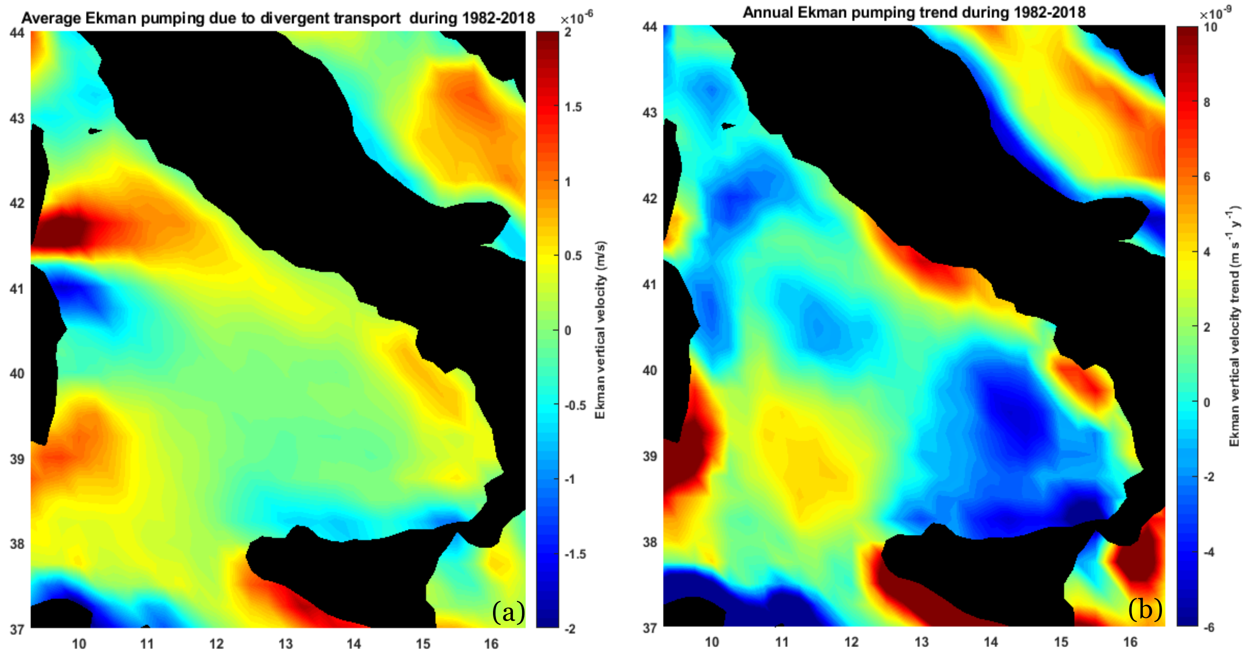

Fig. S5) Average Ekman pumping where positive (red) values indicate upwelling and vice versa.
(b) Annual Ekman pumping trend where negative (blue) values indicate reduced upwelling in the convergence zone and increased downwelling in the divergence zone.

Table S2) Mean heat flux components, their deseasoned standard deviation and Pearson correlation coefficients (r) with the deseasoned net heat flux ($p<0.05$ in bold) indicating that a large fraction of the net heat flux variability is associated with variations of the latent heat loss.

**Component**	**mean**	**std**	**r (NHF)**
Shortwave (SWHF)	181.897	6.831	**0.225**
Longwave (LWHF)	-81.874	4.807	**0.406**
Sensible (SHF)	-11.529	5.744	**0.902**
Latent (LHF)	-81.420	17.173	**0.970**

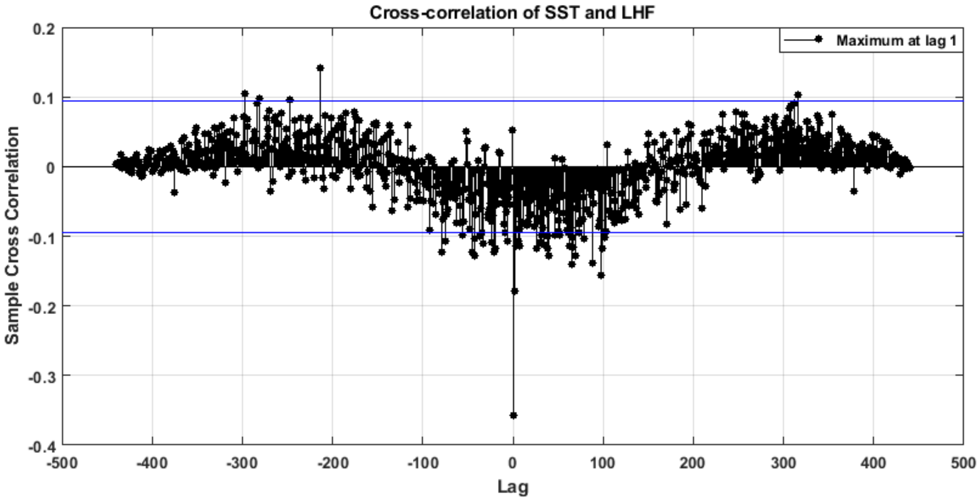

Fig. S6) Lag-lead correlation of SST and latent heat flux representing a nearly symmetric shape centered around lag=1 month, indicating that the coupled variability is characterized by a linear response of the latent heat flux to SST forcing.

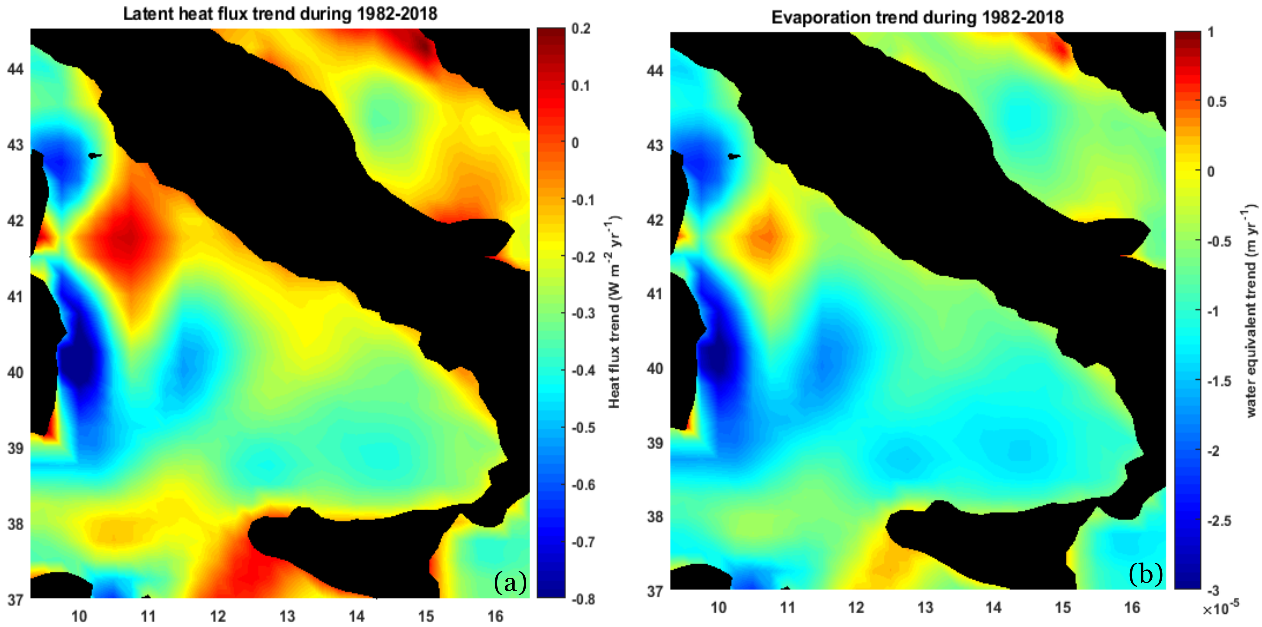

Fig. S7) Spatial trend distribution of (a) latent heat loss and (b) evaporation. Negative values indicate further heat and water loss, respectively. The spatial trend distribution of the latent heat flux is almost identical to that of the net heat flux (not shown), the moisture flux (not shown) and the evaporation. The SST appears to drive the air-sea heat flux variability in the Tyrrhenian Sea over the considered period through latent heat loss variations.

Dominant spatio-temporal features of the SST variability

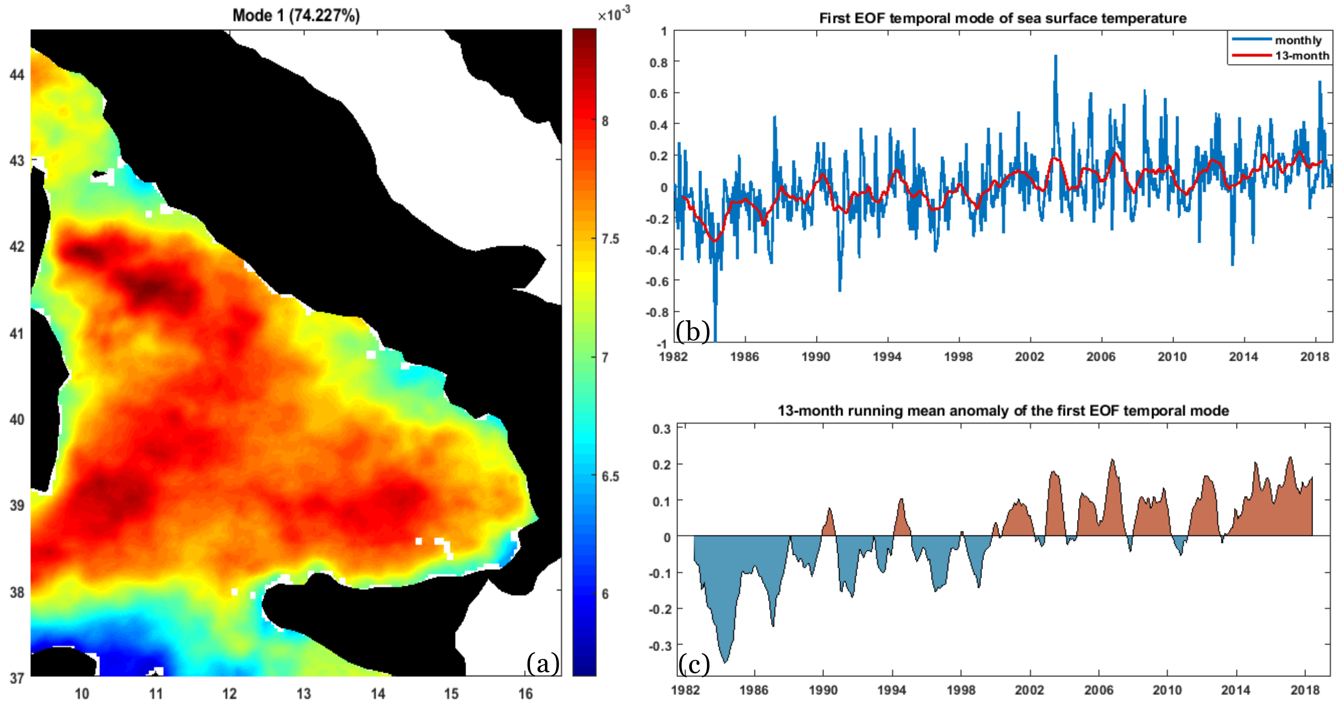

Fig. S8) First EOF of the SST anomaly: (a) spatial amplitude (in °C), (b) normalized temporal mode and (c) 13-month running mean anomaly. The positive values of the spatial amplitude throughout the area indicate an in-phase warming in the entire Tyrrhenian basin, whereas the temporal mode shows strong interannual variability and a trend from a mean negative to a mean positive variation period.

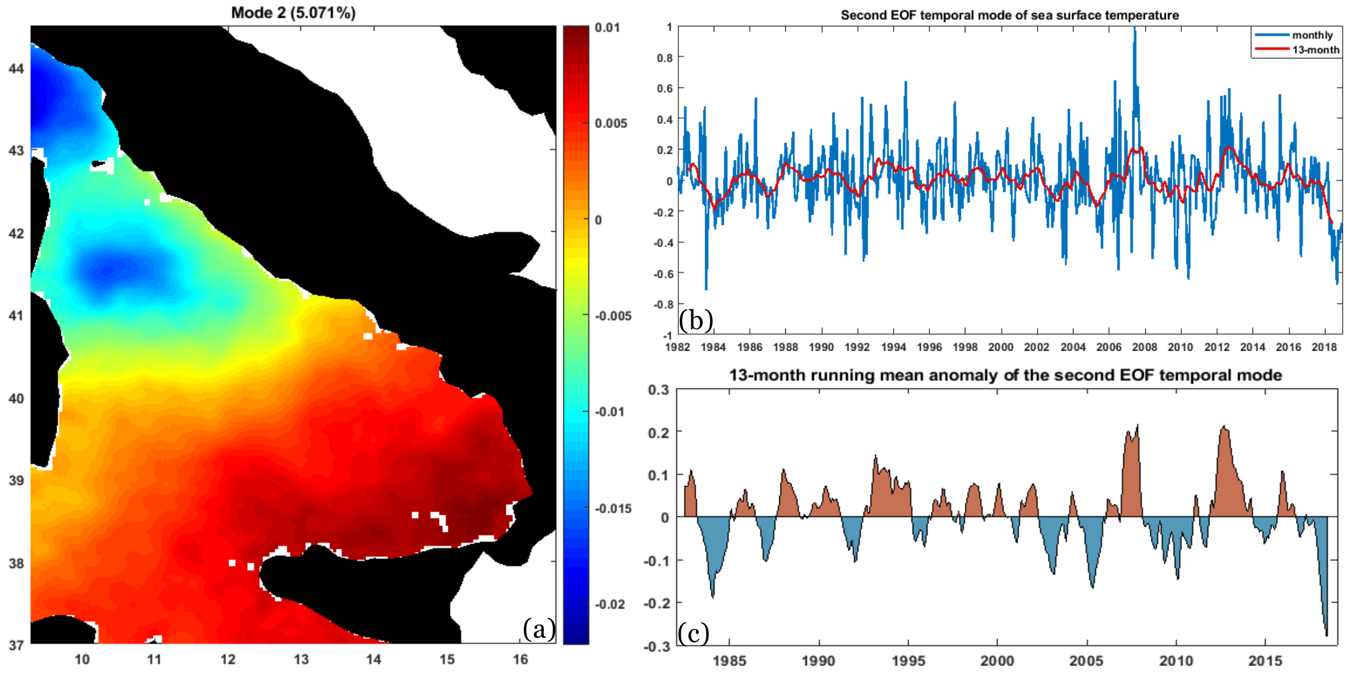

Fig. S9) Second EOF mode: (a) spatial amplitude, (b) temporal mode (normalized) and (c) 13-month running mean anomaly. The principal component showed important both high-frequency and interannual scale variability, whereas the spatial pattern indicated a meridional gradient, with the northern and southern regions of the basin in opposition of phase. This might indicate a dipole oscillation in SST anomalies, where warm anomalies extend across the south-eastern basin, especially between 13 and 16E, and negative SST anomalies extend mainly to the area of the NTC and the Ligurian Sea.

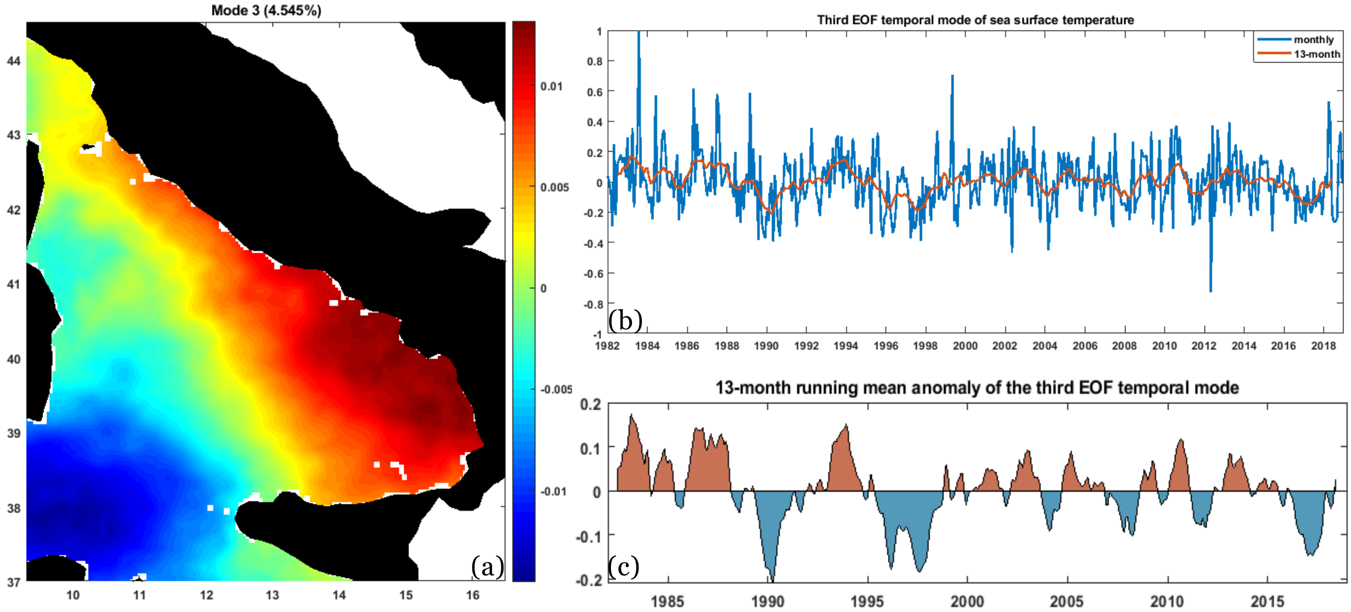

Fig. S10) Third EOF mode: (a) spatial amplitude, (b) temporal mode (normalized) and (c) 13-month running mean anomaly. The third EOF mode explained approximately 4.5% of the total non-seasonal variance. The spatial distribution of this mode represented a zonal gradient with opposite variation peaks between the eastern part of the basin and the western part of the basin, where maximum warm anomalies were observable in the easternmost part. The filtered corresponding principal component time series of the third mode showed significant shifts in the 1990s which coincided with the alternating decrease and increase of basin-average SST during these years.

Large-scale atmospheric and oceanic teleconnection patterns

Table S3) Pearson correlation coefficients between annual normalized basin-averaged SST, the leading SST modes, NAO, AMO, EA and SCAND indexes and their 5-year running means, respectively ($p<0.05$ in bold). The correlation between the AMO index and the SST indicates a link between the SST variability in the Tyrrhenian Sea and the oscillation of the SST in the North Atlantic.

Correlation	AMO	NAO	EA	SCAND
NAO	**-0.504**			
EA	**0.508**	*-0.086*		
SCAND	*-0.228*	*-0.070*	*-0.214*	
SST	**0.589**	*-0.023*	**0.654**	*-0.144*
SST 1 EOF	**0.570**	*-0.026*	**0.649**	*-0.169*
SST 2 EOF	*-0.038*	*-0.201*	*-0.035*	*-0.243*
SST 3 EOF	*-0.238*	*0.021*	*-0.014*	*0.151*

Correlation	AMO 5	NAO 5	EA 5	SCAND 5
NAO 5	**-0.526**			
EA 5	**0.781**	*-0.119*		
SCAND 5	*-0.070*	**-0.437**	*-0.257*	
SST 5	**0.907**	*-0.288*	**0.811**	**-0.168**
SST 1 EOF 5	**0.899**	*-0.287*	**0.806**	*-0.170*
SST 2 EOF 5	*-0.199*	*-0.233*	**-0.430**	*0.135*
SST 3 EOF 5	**-0.474**	*-0.015*	*-0.277*	**0.383**

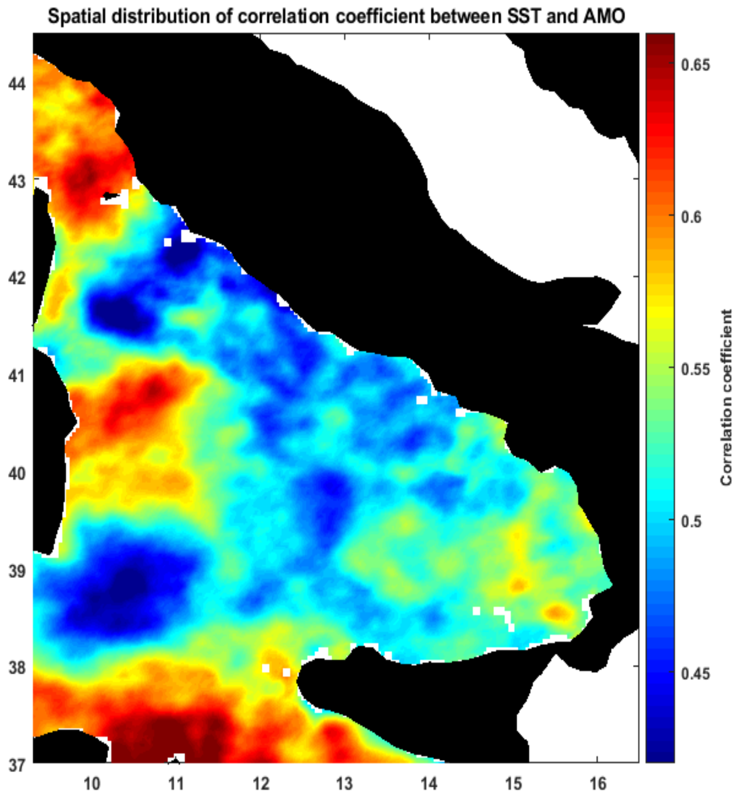

Fig. S11) Spatial distribution of the correlation coefficient between the SST variability and the AMO indicating similar spatial patterns with the overall warming trend (Fig. S1) of the Tyrrhenian Sea.

Supplementary References

1. Pastor, F., Valiente, J. & Palau, J. Sea Surface Temperature in the Mediterranean: Trends and Spatial Patterns (1982–2016). *Pure Appl. Geophys.* **11**, 4017-4029 (2018).
2. Shaltout, M. & Omstedt, A. Recent sea surface temperature trends and future scenarios for the Mediterranean Sea. *Oceanologia* **56** (3), 411-443 (2014).
3. Pisano, A., Marullo, S., Artale, V., Falcini, F., Yang, et al. New Evidence of Mediterranean Climate Change and Variability from Sea Surface Temperature Observations. *Remote Sensing*, **12** (1), 132 (2020).
4. Nykjaer, L. Mediterranean Sea surface warming 1985-2006. *Clim. Res.* **39**, 11-17 (2009).
5. Skliris, N., Sofianos, S., Gkanasos, A., Mantziafou, A., Vervatis, V., et al. Decadal scale variability of sea surface temperature in the Mediterranean Sea in relation to atmospheric variability. *Ocean Dynamics* **62** (1), 13-30 (2012).
6. Criado-Aldeanueva, F., Del Río Vera, J. & García-Lafuente, J. Steric and mass induced sea level trends from 14 years altimetry data. *Global and Planetary Change* **60**, 563-575 (2008).
